# Computer-Assisted Classification Patterns in Autoimmune Diagnostics: The AIDA Project

**DOI:** 10.1155/2016/2073076

**Published:** 2016-03-03

**Authors:** Amel Benammar Elgaaied, Donato Cascio, Salvatore Bruno, Maria Cristina Ciaccio, Marco Cipolla, Alessandro Fauci, Rossella Morgante, Vincenzo Taormina, Yousr Gorgi, Raja Marrakchi Triki, Melika Ben Ahmed, Hechmi Louzir, Sadok Yalaoui, Sfar Imene, Yassine Issaoui, Ahmed Abidi, Myriam Ammar, Walid Bedhiafi, Oussama Ben Fraj, Rym Bouhaha, Khouloud Hamdi, Koudhi Soumaya, Bilel Neili, Gati Asma, Mariano Lucchese, Maria Catanzaro, Vincenza Barbara, Ignazio Brusca, Maria Fregapane, Gaetano Amato, Giuseppe Friscia, Trai Neila, Souayeh Turkia, Haouami Youssra, Raja Rekik, Hayet Bouokez, Maria Vasile Simone, Francesco Fauci, Giuseppe Raso

**Affiliations:** ^1^Faculté des Sciences, Université de Tunis El Manar, 1088 Tunis, Tunisia; ^2^Dipartimento di Fisica e Chimica, Università Degli Studi di Palermo, 90128 Palermo, Italy; ^3^Hôpital Charles Nicolle, 1006 Tunis, Tunisia; ^4^Institut Pasteur, 1002 Tunis, Tunisia; ^5^Hôpital Ariana, 2080 Tunis, Tunisia; ^6^Ospedale S. Antonio Abate, 91016 Trapani, Italy; ^7^Ospedale Buccheri La Ferla Fatebenefratelli, 90123 Palermo, Italy; ^8^Ospedale Civico, 90121 Palermo, Italy; ^9^Ospedale di Sciacca, 92019 Agrigento, Italy; ^10^CyclopusCAD srl, 90128 Palermo, Italy

## Abstract

Antinuclear antibodies (ANAs) are significant biomarkers in the diagnosis of autoimmune diseases in humans, done by mean of Indirect ImmunoFluorescence (IIF) method, and performed by analyzing patterns and fluorescence intensity. This paper introduces the AIDA Project (autoimmunity: diagnosis assisted by computer) developed in the framework of an Italy-Tunisia cross-border cooperation and its preliminary results. A database of interpreted IIF images is being collected through the exchange of images and double reporting and a Gold Standard database, containing around 1000 double reported images, has been settled. The Gold Standard database is used for optimization of a CAD (Computer Aided Detection) solution and for the assessment of its added value, in order to be applied along with an Immunologist as a second Reader in detection of autoantibodies. This CAD system is able to identify on IIF images the fluorescence intensity and the fluorescence pattern. Preliminary results show that CAD, used as second Reader, appeared to perform better than Junior Immunologists and hence may significantly improve their efficacy; compared with two Junior Immunologists, the CAD system showed higher Intensity Accuracy (85,5% versus 66,0% and 66,0%), higher Patterns Accuracy (79,3% versus 48,0% and 66,2%), and higher Mean Class Accuracy (79,4% versus 56,7% and 64.2%).

## 1. Introduction

Autoimmune diseases are due to a reaction of the immune system to self-antigens, occurring through tolerance breakage. The targeted antigens could be common to all kinds of cells or organ specific, and their recognition by humoral or cellular immune effectors could lead to diversified symptoms, depending on pathology [1–3].

There are over 80 different AID, and collectively they are amongst the most prevalent diseases in the US, affecting at least 7% of the population. Because most AID are chronic and incurable, from a public health perspective they constitute a major health problem which, besides causing individual suffering, has high societal costs [4]. These diseases can affect people of all ages and both sexes, with a higher frequency in women of child-bearing age. The autoimmune diseases are multifactorial, and their risk factors are genetic and environmental. The combination of risk factors may vary from one population to another, generating different epidemiological profiles.

Presence of autoantibodies in patient sera has in itself a value of diagnosis, and the ascertaining of their titer and specificity helps to confirm the autoimmune disease and its follow-up. The search of autoantibodies in sera is based on a routine technique performed by Immunologists and on Indirect ImmunoFluorescence (IIF) [5].

The IIF is the Gold Standard for the diagnosis of autoimmune diseases. IIF is a test having high sensitivity, but only analytical and not diagnostic specificity, since the positivity for ANA does not automatically confirm the presence of autoimmune disease; indeed the ANA may be present even in healthy subjects. Furthermore, the quality of the response is strongly influenced by Reader's experience, by the quality of reagents used for testing (characteristics of the cell substrate or fluorochrome-labeled anti-human immunoglobulins used), and by other local factors. As regards the methods immunochemical alternatives, they have the major advantage of being more easily automated and do not require great expertise in interpretation of the results. By contrast the number of antigenic specificities reportable in the test is certainly lower than that detectable on Hep-2 cells and also the integrity of the antigenic epitopes theoretically detectable is not always preserved [6]. The binding of autoantibodies on HEp-2 cells is revealed by fluorescent antibodies to human immunoglobulin. The fluorescence pattern observed on the microscope (Homogeneous, Fine Speckled, Coarse Speckled, Nucleolar, Centromere, Nuclear Dots, etc.) is specific according to the nature of the self-antigen and its location in the cell.

The main disadvantage of IIF technique is its subjectivity in the interpretation of results, highly depending on the experience of the operator. The difficulty of IIF diagnosis technique is related to the distinction of very similar fluorescence patterns (such as Fine Speckled and Coarse Speckled patterns) and to the subjectivity of the observer. For that reason, two Senior Immunologists (double reading) with strong experience in fluorescent image interpretation are quite often needed. However, this condition is not respected in all immunology laboratories involved in diagnosis.

The introduction of new modern approaches, based on computer systems, is an economic and effective support for the diagnosis of autoimmune diseases [7, 8].

Nowadays the need within the scientific community for a large database of IIF images reported out by medical experts is on the increase. Its use could be related to various purposes: training of young Immunologists, epidemiological studies, diagnosis, and so forth. Storing, processing, and sharing such data necessarily require computer techniques [9]. Moreover, computing support is needed in order to avoid difficulties of IIF images interpretation. As already happening with other medical areas facing the same kind of problems (e.g., Radiology), the second Reader could be replaced by a CAD (Computer Aided Detection) solution [10, 11]. In this paper, computer-assisted diagnosis on IIF images, as performed in the AIDA Project, is presented and discussed.

## 2. The AIDA Project

The AIDA (autoimmunity: diagnosis assisted by computer) Project has been financed by a EU cross-border cooperation Italy-Tunisia, involving four teams in Sicily and four teams in Tunis, as presented below.

The aim of the AIDA Project is the applications of ICT for the analysis and interpretation of IIF images.

The AIDA Project has two main specific objectives:

- the creation of a large database of IIF images, interpreted with the contribution of Italian and Tunisian hospitals;
- the evaluation of the added value of CAD, with cooperation from an Immunologist as a second Reader in detection of autoantibodies.

These specific objectives are being achieved through the production of a set of expected results:

- the improvement of organizational and decisional processes of public health policies that will lead to construction of an interinstitutional horizontal partnership (between health facilities) and vertical partnership (region/province/university/ASP),
- the creation of a database of IIF tests images including medical report data,
- application and validation of computer expert systems to support IIF diagnosis in all hospitals involved,
- cooperation between Sicily and Tunisia in research and training within the field of immunology and medical imaging,
- conduction of epidemiological studies in autoimmune diseases within regions geographically close though traditionally different in terms of customs and lifestyle,
- increase in employment opportunities within health applied ICT, such as distribution activities, production, and services within this area of expertise, both in Tunisia and in Sicily.

## 3. Materials and Methods

### 3.1. IIF Technique and Protocol

As reported in this paper, within the context of the project, the IIF technique has been applied to HEp-2 cells using patients sera selected from immunology laboratories for detection of autoantibodies. Manufacturers of kits and instruments employed for ANA testing in AIDA were different site-to-site; the following automated systems solution for the processing of Indirect ImmunoFluorescence tests has been used: IF Sprinter from Euroimmun, NOVA from INOVA diagnostic, Helios from Aesku.

Serial dilutions were carried out and the dilution of 1/80 was considered positive. After incubation of the 1/80 serum dilution, bound antibodies are revealed by fluorescent antibodies to human immunoglobulin. HEp-2 images have been acquired by means of a unit consisting of a fluorescence microscope (40-fold magnification) coupled with a 50 W mercury vapor lamp and a digital camera. The camera has a CCD sensor equipped with pixel size that equals 3.2 *μ*m × 3.2 *μ*m. The images have 24-bit color depth and are stored in low compression jpeg format. The negativity or positivity of the serum is established along with the fluorescence pattern (Homogeneous, Fine Speckled, Coarse Speckled, Nucleolar, Centromere, Nuclear Dots, etc.) reflecting the autoantibodies specificity. One image has been taken for negative sera and three images have been taken for positive sera.

### 3.2. Database

Using a uniform approach, three Tunisian immunology services (Pasteur, Charles Nicolle, and Ariana) and four Sicilian ones (ASP-Trapani, Buccheri La Ferla, Civico, and Sciacca) contributed to collection of images of IIF test on HEp-2 cells. These images correspond to the routine IIF technique performed in the different hospitals for autoimmune diseases diagnosis and were thus reported by Senior Immunologists. A total of 5762 sera of patients addressed for diagnosis of autoimmune disease were involved in this study, as indicated in Table 1. Each image and related report was stored in a common database created in the context of AIDA Project. The database stored the contents of two years' activity and reached a total amount of 14393 stored images. This number is very high compared to public IIF images databases available worldwide (public dataset can be downloaded after proper registration, on site: http://i3a2014.unisa.it/).

A portion of the database utilized is composed of negative images (1446, corresponding to approximately 10%), that is, images whose fluorescence values are not to be associated with autoimmunity problems; 10% of negative cases were considered a satisfactory percentage, in terms of statistics and value purposes, for both Immunologists and the CAD.

**Figure 1:**
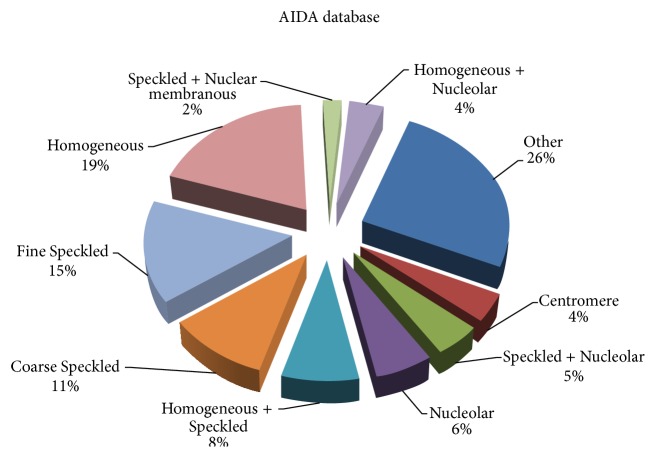
Distribution of IIF patterns in the AIDA database (number of images 14393).

The remaining images were classified into different patterns (according to the physician reporting); the distribution rates are shown in Figure 1. In a second step, 6974 images were anonymously exchanged between partners in full respect of ethics and for the purpose of blind double reporting by Senior or Junior Immunologists. The Junior Immunologists are Ph.D. students (3) and are fundamental Immunologists (1). The four young Immunologists (*Juniors*), all coming from the Tunis Faculty of Sciences, were involved as Readers of images already reported by the experts (*Seniors*). In this first study on the benefits of using the CAD for the correct interpretation of IIF test, we wanted to investigate how the system can be a helpful tool to Junior Immunologists.

### 3.3. Statistics Calculation

The Accuracy and the Mean Class Accuracy (MAC) are adopted in this work as measures of the performance [12].

Let CCR_*k*_ be the correct classification rate for class *k* determined as follows:

(1) $$\begin{array}{c} {CCR}_{k}=\frac{T_{k}}{N_{k}}, \end{array}$$

where *T*_*k*_ is the number of correct identifications of class *k*, while *N*_*k*_ is the total number of elements of class *k*. The Accuracy is defined by

(2) $$\begin{array}{c} Accuracy=\frac{\sum_{k}^{}CCR_{k}\cdot N_{k}}{\sum_{k}^{}N_{k}}. \end{array}$$

The MAC is determined by

(3) $$\begin{array}{c} MAC=\frac{1}{k}\underset{k}{\sum }CCR_{k}. \end{array}$$

Chi-square test was used to check the relationship between the two classification systems. To test the strength of agreement, interrater agreement statistics was conducted [13]. McNemar test was performed to check the difference for paired proportions. *p* values of less than 0.05 were considered significant.

### 3.4. CAD Immuno

Computer Aided Diagnosis (CAD) systems are widely used for different tasks within medicine such as second reading, increasing the diagnosis speed, and training physicians for special task. One of such systems is proposed in recent days for automatic HEp-2 images classification, which is important for detection of antibodies in human serum. In the AIDA Project the* CyclopusCAD Immuno* software was used, powered by CyclopusCAD ltd., a spin-off of University of Palermo [14–18]. It consists of a computer system for image analysis using artificial intelligence methods, of which some are proprietary [19, 20]. The performance comparison between CyclopusCAD Immuno and several automated systems for IIF analysis was presented in a recent paper by Gorgi et al. [21].

### 3.5. Medical Context

For each well the diagnostic procedure consists of fluorescence intensity classification, mitotic cells recognition, and staining pattern classification for interphase cells. Interestingly, all these steps can be formulated as pattern recognition problems. The medical doctor evaluates the fluorescence intensity of the sample classifying it into three classes, named negative, intermediate, and positive.

**Figure 2:**
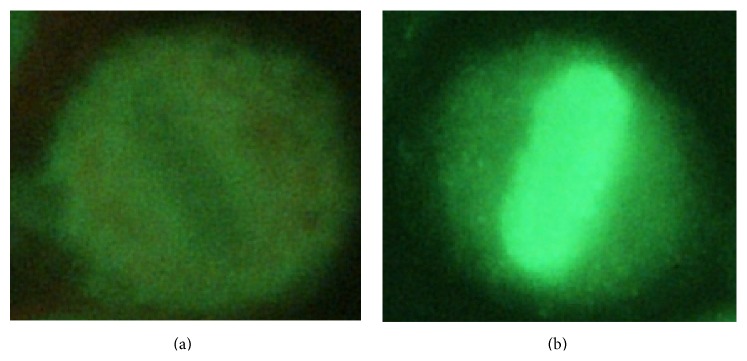
Examples of negative and positive mitosis ((a) and (b), resp.).

Such classification is important since it affects staining pattern identification for interphase cells, which is performed only on positive or intermediate samples. Mitotic cells recognition aims at verifying the presence of at least a cell in mitosis within the image analyzed. Mitotic cells may exhibit two fluorescence patterns. The first, named as negative mitosis, is characterized by a fluorescent cell body while the collapsed chromosomes mass, located in the middle part of the cell, does not exhibit a fluorescent patter or has a weak fluorescence. With the second pattern, reported as positive mitosis, we observe the opposite situation: that is, the cell body is weakly fluorescent or nonfluorescent, while the chromosomes mass is fluorescent.  Figure 2 shows examples of negative and positive mitosis. The third step of the IIF diagnostic procedure aims at recognizing the staining pattern of interphase cells. HEp-2 samples with nonnegative fluorescence intensity may reveal different staining patterns, relevant for diagnostic purposes. The patterns classification is performed by the topographic survey of the nuclear fluorescence during the cell cycle. In particular, the chromatin positivity in the phases of the cycle preceding the cell division indicates the possible presence of a Homogeneous pattern. However, this positivity of the chromatin must be differentiated with respect to the case where the chromatin positivity is present in a Centromere pattern. On the contrary, the chromatin negativity is used to direct the classification to those patterns characterized by such negativity (Speckled, Nucleolar) (see also http://www.anapatterns.org/).

**Figure 3:**
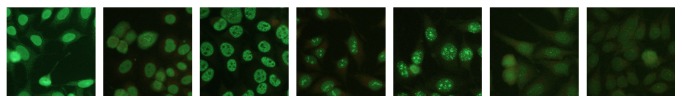
IIF images with different staining patterns (from left to right: Homogeneous, Fine Speckled, Coarse Speckled, Nucleolar, Centromere, Nuclear Dots, and Nuclear Pore Complex).

The most frequent and clinically useful staining patterns are represented in Figure 3.

It is worth observing how the mitotic cell recognition step is important for two reasons: first, the presence of at least one mitotic cell confirms with medical doctors the correctness of well preparation, so that the well is discarded if no cell in mitosis is detected; second, the information on the fluorescence pattern of mitotic cells can be used to improve the ability to discriminate between similar stainings of interphase cells [22].

### 3.6. CAD Workflow

**Figure 4:**
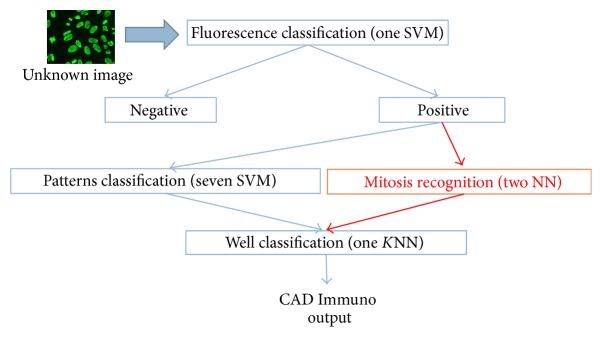
CyclopusCAD Immuno working flow: the system aims to reproduce the operations flow made by Immunologist and described in Section 3.5, by making a classification of fluorescence only for nonnegative images, and by operating a patterns classification and a mitosis classification; it will then be using the results of these classifications to provide a final output.

The CAD Immuno is able to identify, on IIF images, the fluorescence intensity and the fluorescence pattern. In particular, the analysis of fluorescence images for the positive/negative detection is carried out using a Support Vector Machine (SVM) classifier. The system is able to recognize the following fluorescence patterns: Homogeneous, Fine Speckled, Coarse Speckled, Nucleolar, Centromere, Nuclear Dots, and Nuclear Pore Complex. The system searches and classifies positive mitosis and negative mitosis within the image. The classification of mitosis occurs by using two Neural Network (NN) classifiers. The final decision-making process for the detection of fluorescence pattern is achieved by using a *K*-Nearest Neighbors (*K*-NN) classifier, having nine inputs (seven outputs of patterns classifiers and two mitosis classifiers).  Figure 4 shows the CAD Immuno working flow.

In several multiclass classification problems, it is preferable to use a number of classifiers equal to the number of classes and each classifier is trained to discriminate a class from all the others (binary approach) [23]. In this system, in addition to differentiating the classification stage by implementing seven classifiers for seven classes of staining patterns, the preprocessing, segmentation, and feature extraction steps are differentiated as well. The CAD Immuno here presented adopts a nonstandard pipeline for supervised image classification.

**Figure 5:**
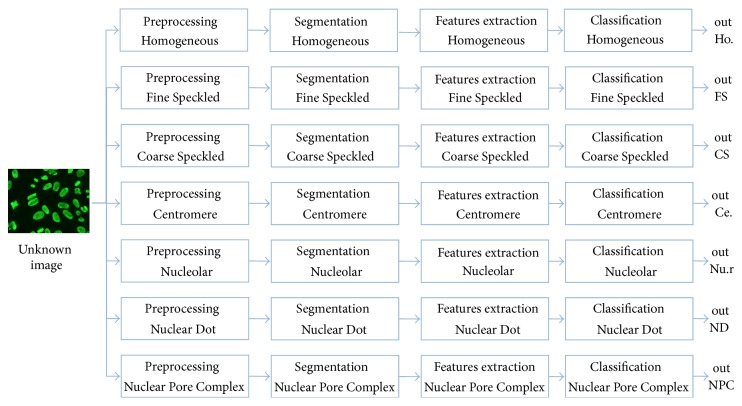
Pipeline of patterns classification method: the generic new image is simultaneously processed by seven processes, thus obtaining seven separate outputs showing how the cell resembles each of the 7 classes analyzed in this work; as an example, out Ho. represents the degree of similarity between the unknown image and the Homogeneous images.

Figure 5 shows the flow of operations adopted in this work; the generic new image is simultaneously processed by seven processes, obtaining seven separate outputs representing how the cell resembles each of the 7 classes analyzed in this work. The choice of methods, features, and parameters was performed automatically, using the Mean Class Accuracy (MCA) as a “figure of merit.” The parameters involved are tuned using a cross validation scheme. The main benefit of this pipeline consists in offering of a good explanatory faculty, based on an easily explainable principle.

### 3.7. Features

Cells patterns analysis clearly shows that differences between classes are mainly based on the presence and distribution of bright/dark structures: numbers, sizes (areas), intensity, and colors. It seems natural to use features providing an analysis of texture and more specifically features able to deal with bright/dark speckle-like structure description.

Different staining patterns can be characterized by a limited set of attributes describing the spatial relationships between pixels values and main image variations occurring in each cell type; this information is generally obtained by mean of textural analysis techniques. These techniques can be grouped into two major categories: (i) statistical methods describing the distribution of grey-levels in the image and (ii) frequency domain measurements of image variations [24–26]. To achieve the objective of robust classification, we combined several discriminative visual features known to be effective for cell classifications with a robust and scalable multiclass boosting. All classifiers, developed in this project by CyclopusCAD, use a large number (108) of extracted features, able to fully characterize the HEp-2 cells. In more detail, four quantization levels [27] were analyzed (256, 128, 64, and 32), and for each of them the following 27 features have been extracted [28].

*(i) Intensity Based Features (9)*. They include mean value, standard deviation, ratio of standard deviation to the mean value, entropy, moment of inertia, skewness, kurtosis, and entropy of the contours gradient.

*(ii) Geometry-Based Features (8)*. They include mean radius, standard deviation of radius, maximum radius, ratio of the standard deviation to the mean value, circularity, anisotropy, fractal index, and eccentricity.

*(iii) Shape-Morphological-Based Features (8)*. They include area, perimeter, convex area, convex deficiency, solidity, compactness, roundness, and Euler's number.

*(iv) Descriptors-Based Features (2)*. They include entropy of HOG (Histogram of Oriented Gradients) and entropy of HAG (Histogram of Amplitude Gradients).

## 4. Results and Discussion

In order to evaluate the concordance level, a subsample of 589 wells (each of them with 3 images), reported by two Senior Immunologists, was analyzed. The results are shown in Table 2 and revealed a concordance level around 71% (Cohen's *K* statistic was 0.64). The maximum level of concordance has been obtained for the Centromere pattern. The lower concordance level is observed with the Fine Speckled pattern. This concordance level represents the difficulty of interpretation of IIF images and the obvious need for a double reading and for Gold Standard. Taking only images into account, with a concordance of interpretation between the two Senior Immunologists, a subsample database was then extracted containing 1006 images, representing all types of patterns (302 wells corresponding to 906 images) and including negative tests (100 images).

**Figure 6:**
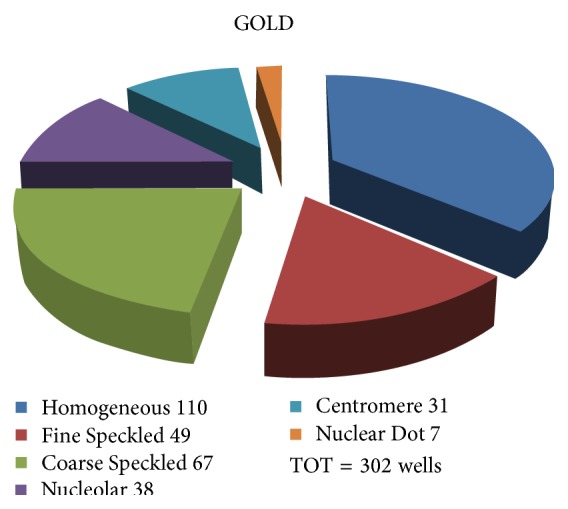
Distribution of IIF patterns in the Gold Standard database (number of images 1006 and number of wells 302).

In this Gold Standard database, the distribution of the different patterns is given in Figure 6. The overall approach is resumed in Table 3.

Our objective is to evaluate the added value of the CAD used as a second Reader. We first tested CAD performance* stand-alone* in comparison with two Junior Immunologists. Four young fundamental Immunologists (*Juniors*) were involved as Readers of images already reported by the experts (*Seniors*). Before being compared to the CAD, the reporting concordances of these Junior Readers were compared with each other and to those of a Senior Immunologist. As shown in Table 4, and as expected, the concordance level between Junior and Senior Immunologists was lower than that observed when comparing only Seniors. The only exception to this is with Reader 4, who is a more experienced fundamental Immunologist but has never been involved in diagnosis.

We also compared the Junior Readers with one another. We looked at two pairs: Juniors 1 and 2 against Juniors 3 and 4. The results indicate, for each pair, a concordance level that is near to the mean concordance of a Senior.

In another step we assessed the performance of two Juniors reporting on Gold Standard wells (each of them with 3 images). Accuracy was established by considering intensity on one hand and patterns on the other.

We then compared this performance to that of the CAD, which gave better results than Junior Immunologists, as shown in Tables 5 and 6.

Compared with the two Readers, CyclopusCAD Immuno software showed higher Intensity Accuracy (versus Junior 1  *p* = 0.016, versus Junior 2  *p* = 0.016), higher Patterns Accuracy (versus Junior 1  *p* < 0.0001, versus Junior 2  *p* = 0.1), and higher MAC (versus Junior 1  *p* = 0.0026, versus Junior 2  *p* = 0.057).

In a last step we assessed the performance improvement of the Junior Immunologist reports with the support of the CAD. The increase in accuracy, reported in Table 7, is observed particularly for Junior Reader 1.

With respect to the performance of Junior 1,

(i) Intensity Accuracy varied from 66% to 76%, showing an increase (*p* = 0.21);
(ii) Patterns Accuracy varied from 48% to 69.5% and was significantly increased (*p* = 0.002);
(iii) MAC varied from 56.7% to 73.9% and was significantly increased (*p* = 0.02);
(iv) Cohen's *K* varied from 0,36 to 0,61.

With respect to the performance of Junior 2,

(i) Intensity Accuracy showed no variation;
(ii) Patterns Accuracy varied from 66.2% to 68.2%, showing a low significance increase (*p* = 0.81);
(iii) MAC varied from 64.2% to 66.3, showing a low significance increase (*p* = 0.79);
(iv) Cohen's *K* varied from 0,58 to 0,60.

## 5. Conclusions

In this paper the AIDA Project and its preliminary results are presented. The AIDA Project initiative in Italy and Tunis suggests that a joint effort by Health Professionals, Scientific Societies, and patients' associations can make a difference. Even before its closure the preliminary results produced by the AIDA Project are very encouraging. The size of the database, with around 14500 images and each with their respective report, is the biggest within the field of Indirect ImmunoFluorescence applied to autoimmune diseases diagnosis in the world. Additionally we have compiled around 1000 images with two concordant reports established by immunology experts and including different patterns, which have composed our first Gold Standard database. This has been the basis for “learning” of the* CyclopusCAD Immuno* software. This Gold Standard database has proven to be of great interest and has been considered for use as reference to evaluate Junior and Fundamental Immunologists and CAD performance. The automatic CAD system used and optimized in the AIDA Project has been described and its performance was evaluated.

In this work the results in terms of performance of Junior Immunologists, CAD, and Immunologists with the aid of CAD have also been reported. The CAD system showed higher Intensity Accuracy (85,5% versus 66,0% and 66,0%), higher Patterns Accuracy (79,3% versus 48,0% and 66,2%), and higher MAC (79,4% versus 56,7% and 64.2%).

Another objective of this project is to evaluate the added value of the CAD used as a second Reader. The analysis conducted here showed that the CAD support improves the performances of Junior Immunologists up to as much as 45%, as highlighted by the comparisons reported on Tables 5 and 7.

Considering the concordance level between Fundamental Immunologists evaluated at 71%, the CAD seems to bring the same level of performance. Indeed, when compared to Gold Standard, the CAD showed a mean accuracy of 85% for intensity evaluation and 79% for pattern recognition.

With these preliminary results, we can conclude that the objectives of the project are being reached with the demonstration that the CAD shows a higher performance than Junior Immunologists and equivalent results with Immunology Experts. In a further step, the Gold Standard database should be enriched, including the addition of more double reported and triple reported images. With such a high number of images of high-quality reporting, we would be able to improve the CAD performance and our quality assessment approach in order to offer a product of high confidence that could be used as second Reader to student learning and to remote diagnosis.

## Figures and Tables

**Table 1:** Number of sera and images.

Number of patients	Results of IIF test	Number of selected images	Total images
5762	4316 Positive	12947	14393

**Table 2:** Level of concordance between two Senior Immunologist Readers of 589 wells.

	Senior 1								
		Negative	Homog.	Fine S.	Coarse S.	Nucleol.	Centrom.	Dot	TOT
Senior 2	Negative	**117**	6	10	5	3			141
	Homog.	5	**110**	15	3	1			134
	Fine S.	26	22	**49**	24	1			122
	Coarse S.	23	4	20	**67**				114
	Nucleol.	1				**38**			39
	Centrom.						**31**		31
	Dot					1		**7**	8
	TOT	172	142	94	99	44	31	7	**589**

**Table 3:** Number of IIF images in AIDA database: with one or two reports among which a Gold Standard sample with concordant reporting was extracted.

	Images		
	With 1 report	With 2 reports	*Gold Standard*
Positive	12947	6274	906
Negative	1446	700	100
Total	**14393**	6974	1006

**Table 4:** Percent of reporting concordance of Junior Immunologist versus Senior or Junior Immunologist.

Concordance% of Juniors versus Seniors				
Junior Readers	1	2	3	4
Number of wells	117	169	174	141
Concordance	37,6%	53,2%	42,5%	72,3%
Mean	45,8%		57,4%	

Concordance% of Junior versus Junior				
Juniors pair	1 versus 2		3 versus 4	

Number of wells	265		219	
Concordance	46,8%		68,5%	

**Table 5:** Comparison of CAD and Junior reporting using Gold Standard images as reference.

Readers	Intensity	Patterns		
	Accuracy	Accuracy	Mean accuracy	Cohen's *K*
Junior 1	66,0%	48,0%	56,7%	0,36
Junior 2	66,0%	66,2%	64,2%	0,58
CAD	**85,5%**	**79,3%**	**79,4%**	**0,36**

**Table 6:** Accuracy and mean accuracy of CAD compared to Gold Standard used as reference.

CAD	Homog.	Fine S.	Coarse S.	Nucleol.	Centrom.	Dot	Other	ACC	MAC
Homog.	**81,1%**	9,5%	6,8%	2,7%	0,0%	0,0%	0,0%	**79,3%**	**79,4%**
Fine S.	6,5%	**54,8%**	16,1%	19,4%	3,2%	0,0%	0,0%		
Coarse S.	0,0%	9,6%	**80,8%**	3,8%	3,8%	1,9%	0,0%		
Nucleol.	3,3%	10,0%	0,0%	**86,7%**	0,0%	0,0%	0,0%		
Centrom.	3,4%	3,4%	0,0%	3,4%	**89,7%**	0,0%	0,0%		
Dot	0,0%	0,0%	16,7	0,0%	0,0%	**83,3%**	0,0%		
Other	0,0%	0,0%	0,0%	0,0%	0,0%	0,0%	**0,0%**		

**Table 7:** Accuracy of Junior reporting with the support of the CAD using Gold Standard images as reference.

Readers	Intensity	Patterns		
	Accuracy	Accuracy	Mean accuracy	Cohen's *K*
Junior 1	76,0%	69,5%	73,9%	0,61
Junior 2	66,0%	68,2%	66,3%	0,60

## Disclaimer

This document was produced with the financial assistance of the European Union in the frame of the ENPI CT Italy-Tunisia 2007–2013. The views expressed herein can in no way be taken to reflect the official opinion of the European Union. Sole responsibility for the views, interpretations, or conclusions contained in this document lies with the authors. Neither the European Commission nor Management Structures of the Program can be held responsible for the accuracy, completeness, or use of the information contained herein.
